# Association Mapping of Yield and Yield-related Traits Under Reproductive Stage Drought Stress in Rice (*Oryza sativa* L.)

**DOI:** 10.1186/s12284-017-0161-6

**Published:** 2017-05-18

**Authors:** B. P. Mallikarjuna Swamy, Noraziyah Abd Aziz Shamsudin, Site Noorzuraini Abd Rahman, Ramil Mauleon, Wickneswari Ratnam, Ma. Teressa Sta. Cruz, Arvind Kumar

**Affiliations:** 1 Plant Breeding Division, International Rice Research Institute (IRRI), DAPO Box 7777 Metro Manila, Philippines; 2 Faculty of Science and Technology, Universiti Kebangsaan Malaysia, 43600 Bangi, Selangor Malaysia; 3 MARDI, Seberang Perai, P.O. Box No. 203, 13200 Kepala Batas, Pulau Pinang Malaysia

**Keywords:** Drought, Genetic diversity, Association mapping, Genes

## Abstract

**Background:**

The identification and introgression of major-effect QTLs for grain yield under drought are some of the best and well-proven approaches for improving the drought tolerance of rice varieties. In the present study, we characterized Malaysian rice germplasm for yield and yield-related traits and identified significant trait marker associations by structured association mapping.

**Results:**

The drought screening was successful in screening germplasm with a yield reduction of up to 60% and heritability for grain yield under drought was up to 78%. There was a wider phenotypic and molecular diversity within the panel, indicating the suitability of the population for quantitative trait loci (QTL) mapping. Structure analyses clearly grouped the accessions into three subgroups with admixtures. Linkage disequilibrium (LD) analysis revealed that LD decreased with an increase in distance between marker pairs and the LD decay varied from 5–20 cM. The Mixed Linear model-based structured association mapping identified 80 marker trait associations (MTA) for grain yield (GY), plant height (PH) and days to flowering (DTF). Seven MTA were identified for GY under drought stress, four of these MTA were consistently identified in at least two of the three analyses. Most of these MTA identified were on chromosomes 2, 5, 10, 11 and 12, and their phenotypic variance (PV) varied from 5% to 19%. The *in silico* analysis of drought QTL regions revealed the association of several drought-responsive genes conferring drought tolerance. The major-effect QTLs are useful in marker-assisted QTL pyramiding to improve drought tolerance.

**Conclusion:**

The results have clearly shown that structured association mapping is one of the feasible options to identify major-effect QTLs for drought tolerance-related traits in rice.

**Electronic supplementary material:**

The online version of this article (doi:10.1186/s12284-017-0161-6) contains supplementary material, which is available to authorized users.

## Background

Rice is the primary food source for more than half of the world’s population and contributes 30–50% of the daily caloric intake (Fairhust and Dobermann, 2002). Among different rice ecosystems, rainfed upland and rainfed lowland rice occupy 30% of total rice area but contribute only 21% of total rice production. Drought is one of the most severe climate-related risks for rice production in rainfed areas of Asia and Africa (Pandey, 2007). With limited options for expanding rice area and the existing plateau in the yield potential of irrigated rice, a further increase in rice production has to come from highly vulnerable, less productive drought-prone rainfed lowland and upland rice areas (Khush, 1997). These areas received much less attention during the Green Revolution and even now most of the varieties grown in these areas are ones that were developed for high-input irrigated conditions. These varieties are highly susceptible to the various abiotic and biotic stresses prevalent in low-input rainfed environments. Thus, there is an urgent need to develop climate-smart rice varieties with multiple abiotic and biotic stress tolerance, and with improved grain quality and high yield potential that are suitable for rainfed areas (Kamoshita et al. 2008; Lafitte et al. 2004).

Rapid and precise exploitation of the abundant genetic diversity available within rice germplasm is highly critical to ensuring sustainable rice production and global food security in the ever-changing climatic conditions (McCouch et al. 2012; Voss-Fels and Snowdon 2016). The recent advances in rice biotechnological tools have been very helpful in unraveling the genetic basis of complex traits within rice germplasm to identify major genes/QTLs for use in rice breeding (Thomson et al. 2010; Swamy et al. 2014; Swamy and Kumar, 2013). For improving drought tolerance, several major-effect grain yield QTLs under drought have been identified and successfully used in marker-assisted breeding (MAB) (Swamy et al. 2011). But, most of the QTL studies were on biparental or multiparent populations, which are limited by the allelic diversity within the selected parents. In addition, population development is time-consuming and mapping resolution is low (Kumar et al. 2014; Pascual et al. 2016). Also, the drought QTLs identified from different studies and meta-analysis of *qDTYs* have clearly shown that only a few major-effect *qDTYs* have been consistently and repeatedly identified, indicating limited exploration of genetic resources to identify novel major-effect *qDTYs* (Swamy et al. 2011; Kumar et al. 2014).

Marker-assisted pyramiding of *qDTYs* into elite rice varieties has shown that they have synergistic relationships in particular combinations and their effect varies with the genetic background. Introgression of four *qDTYs* into popular mega-variety IR64 and three *qDTYs* in the background of MR219 showed that *qDTYs* have a better effect in different combinations in different genetic backgrounds (Swamy et al. 2013; Noraziyah et al. 2016a). This further emphasizes the urgent need to identify novel *qDTYs* to improve the drought tolerance of a wide range of drought-susceptible rice varieties.

The availability of genome-wide molecular markers, cheaper genotyping services and advances in statistical analysis have made it possible to explore natural populations to identify significant marker and trait associations, also popularly called genome-wide association studies (GWAS) (Korte and Farlow, 2013). Considering the huge genetic diversity available for multiple traits within rice germplasm, GWAS can be a feasible approach to simultaneously map loci for many traits and the improved mapping resolution helps in precisely identifying the genes/SNPs associated with the traits. There are several successful examples of accurate QTL/gene detection using GWAS in rice and other crop species (Huang et al. 2010; Han and Huang, 2013; Wu et al. 2015; Yang et al. 2014; Kumar et al. 2014), but there are only a few association studies for drought-related traits in rice (Vasant 2012; Courtois et al. 2013; Vannirajan et al. 2012; Muthukumar et al. 2015).

The present study was undertaken with the objectives of screening Malaysian rice germplasm for drought tolerance, determining the population structure, doing association mapping of yield and yield-related traits under drought stress and non-stress (NS) conditions, and carrying out reference genome-based analysis of *qDTY* physical regions.

## Results

### Drought Screening

Drought was imposed in the dry season (DS) by draining out all the water one month after transplanting. Data on rainfall and water table depth were recorded in both trials. The rainfall was less than 86.2 mm during the 2011DS and was 264.1 mm during the 2012DS (Additional file 1: Figure S1 and Additional file 2: Figure S2). The water table depth reached less than 100 cm before flowering during both seasons. The results showed that there was moderate to severe drought stress during the reproductive stage of the crop in both seasons. The NS counterpart was completely flooded from transplanting until the crop reached physiological maturity.

### Phenotypic Analysis

Phenotypic traits measured under both NS and drought stress conditions and in both years showed wide variation (Table 1). The overall mean reduction in GY under drought stress compared with NS was 60% in the 2011DS, 51% during the 2012DS and 53% in combined analysis. In general, there was a reduction in PH and delay in flowering under drought stress compared with NS, indicating the effect of drought stress on plant growth. The heritability (H^2^) for DTF and PH was very high: it varied from 84.0% to 86.0% for DTF and from 86.0% to 87.0% for PH under drought stress in different seasons; whereas, under non-stress, it varied from 83.6% to 87.0% for DTF and from 81.7% to 89.0% for PH. For GY, H^2^ varied from 72.0% to 78.0% under drought stress and from 63.0% to 69.0% under non-stress. Table 2 presents the correlation among the traits under drought stress and NS. Nine out of 18 correlations for three traits under both drought stress and NS were found significant. There was a significant negative correlation between DTF and GY under drought stress in both seasons and also in the combined analysis. Also, PH and GY were negatively correlated under drought stress; whereas, under non-stress, the correlation between PH and GY was significant only in 2012. The coefficient of variation (CV) was moderate to high for all three traits under both drought stress and NS, indicating wide variation.

**Table 1:** Various statistical parameters for yield and yield-related traits under non-stress and drought stress

Trait	Year/season	Mean		Range		SD		CV (%)		H^2^ (%)	
		NS	S	NS	S	NS	S	NS	S	NS	S
DTF (days)	2011DS	93.0	95.0	55.0–124.0	60.0–127.0	12.8	12.1	6.0	6.5	87.0	86.0
	2012DS	88.9	96.1	77.0–120.0	65.0–123.0	9.4	10.6	8.4	6.5	83.6	84.0
	Combined	90.9	95.5	60.0–124.0	62.0–124.0	10.9	11.3	7.3	6.5	85.3	83.5
PH (cm)	2011DS	148.0	94.0	68.7–148.7	55.3–121.3	19.5	12.1	7.1	9.5	89.0	87.0
	2012DS	106.8	87.1	64.3–172.0	64.7–129.7	19.9	13.9	15.2	7.8	81.7	86.0
	Combined	101.0	80.9	68.7–154.3	58.8–119.8	19.3	13.3	11.9	8.5	85.3	86.3
GY (Kg/ha)	2011DS	1736.0	687.7	633.8–3689.0	57.0–1577.0	684.6	327.1	34.2	25.6	63.0	72.0
	2012DS	5766.1	2831.5	930.6–10850	100.0–5615.0	2086.5	1161.3	26.4	24.3	69.0	78.0
	Combined	3832.9	1794.9	672.4–10737	98.0–5295.3	2478.2	1339.9	23.5	25.4	65.4	75.4

### Genotypic Analysis

Out of the 125 SSR markers genotyped in the 75 Malaysian genotypes, 119 (95.2%) were found to be polymorphic. The number of SSR markers varied on different chromosomes: with highest number of 21 markers on chromosome 2, followed by 17 markers on chromosome 1, 11 markers each on chromosomes 9 and 12, on other chromosomes number of markers varied from 4 to 9 and lowest of four markers on chromosome 8, with an average density of one marker for every 3 MB. In all, 119 SSR markers amplified 1524 alleles, out of which 482 (31.6%) were in POP1, 483 (31.7%) were in POP2 and 559 (36.7%) were in POP3. Among the different SSR markers, seven were bi-allelic, 17 were tri-allelic and 12 were tetra-allelic, while 77 SSR markers amplified 5–10 alleles, three markers (RM13, RM6374 and RM180) amplified 11 alleles, two markers (RM335 and RM491) amplified 12 alleles and one marker (RM440) amplified a maximum of 13 alleles in the 75 rice genotypes. Additional file 3: Table S1 presents the overall and chromosome-wise allelic frequency, number of alleles, allelic diversity, PIC and number of subpopulation-specific alleles. Allele frequencies, genetic diversity and PIC were highest on chromosome 5 and lowest on chromosome 7. The PIC values varied from 0.026 to 0.840; seven markers (RM21, RM12460, RM12182, RM17524, RM108, RM553 and RM219) had the highest PIC of more than 0.8016. Gene diversity varied from 0.263 to 0.856 and the major allelic frequency varied from 0.227 to 0.987.

### Population Structure and Kinship

**Fig. 1:**
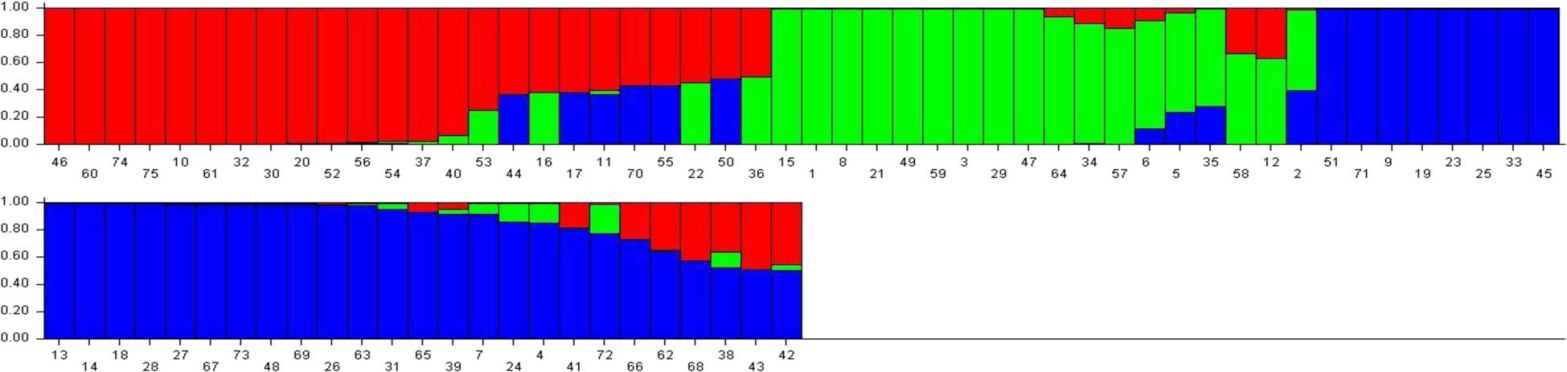
Population structure of 75 accessions based on 119 SSR markers (K = 3)

We performed structural analysis using 119 marker genotypic data from 75 genotypes and the analysis was carried out in ten replications with 500,000 burns. The number of subpopulations (K) was determined based on the posterior probability values (LnP(D) and ΔK). For K = 2 to 10, the values of LnP(D) and ΔK continuously increased in all ten replicates and the highest ΔK of 24.2 was reached when K = 3 with an LnP(D) of−19790.04. Thus, the 75 genotypes were grouped into three subpopulations and the results of the three subpopulations were used for further analysis. The molecular diversity analysis also grouped the genotypes into three clusters, thus supporting the results of the structure analysis. Among the three subpopulations, POP1 consisted of 23 genotypes, POP2 consisted of 18 genotypes and POP3 consisted of 34 genotypes. The fixation index (F_st_) was 0.277, 0.270 and 0.194 in POP1, POP2 and POP3, respectively, and the expected heterozygosity was 0.544, 0.572 and 0.559 in POP1, POP2 and POP3, respectively (Additional file 3: Table S2). Twenty-nine alleles differentiated POP1 and most of them were on chromosomes 1, 2, 4, 5 and 9; in POP2, there were 57 population-specific alleles, with most of them on chromosomes 1, 2, 5, 6, 7, 8 and 11; whereas 61 alleles differentiated POP3, with most of them on chromosomes 1, 2, 7, 9, 11 and 12 (Additional file 3: Table S2). The bar diagram shows the distribution of genotypes within and between the populations (Fig. 1). The allelic frequency divergence between POP1 and POP2 was 0.194, between POP1 and POP3 it was 0.120 and between POP2 and POP3 it was 0.197.

### Linkage Disequilibrium (LD)

**Fig. 2:**
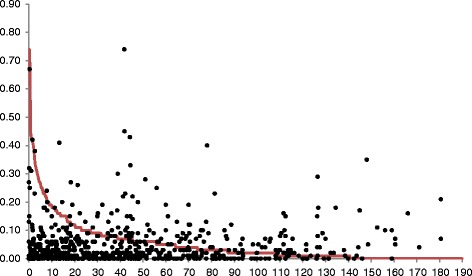
Distribution LD (R^2^) values observed between linked SSR marker pairs as a genetic distance in centiMorgans (cM)

LD analysis in the whole population showed 7072 LD pairs, out of which 2264 (32%) pairs were significant (*P* > 0.05) (Additional file 3: Table S3). There were overall 2071 inter-chromosomal LD pairs and 193 significant intra-chromosomal LD pairs (Additional file 3: Table S3). Chromosomes 1, 2, 3, 6, 9 and 10 had more than 10 intra-chromosomal LD pairs, whereas chromosomes 2, 6, 9, 10 and 12 had more than 200 significant inter-chromosomal LD pairs. Chromosome 2 had the highest number of significant intra- and inter-chromosomal LD pairs, while chromosome 7 had the lowest number. The LD scatter plot showed a reduction in the number of significant LD pairs as the interval distances between marker pairs increased. The LD (*R* ^*2*^) plotted against cM is presented in Fig. 2. There was a sharp decline in LD decay for the linked markers at 5–20 cM. Overall, the *r* ^*2*^ varied from 0.005 to 0.31, with an average *r* ^*2*^ of the locus pairs of 0.03. Significant pairs of linked loci (*r* ^*2*^ *>* 0.2) showed an average distance of 5–20 cM, indicating the presence of a higher number of significant LD blocks in this set of germplasm accessions.

### Association Analysis

The results of association analysis for GY under drought stress and non-stress are presented in Tables 3 and 4, and for DTF and PH in Additional file 3: Tables S4 and S5. In all, there were 198 and 80 significant marker trait associations (MTA) for all three traits identified by GLM and MLM analyses. MTA identified by MLM are further discussed. Among the QTLs indentified by MLM, 7 and 13 of them were for GY under drought stress and NS, respectively. There were five MTA for GY in the 2012DS, four in combined analysis, while there were only three QTLs in the 2011DS. Four MTA for GY under drought stress were consistently identified in at least two of the three analyses. These MTA identified were on chromosomes 2, 5, 6, 10, 11 and 12. The PV of the QTLs varied from 5 to 19; five MTA had more than 10% PV. It is interesting to note that the novel QTL on chromosome 5 was consistently identified in all the three analyses and had highest PV of 19%. Under NS, seven MTA were identified in the 2011DS, five in combined analysis, while only two MTA were significant in the 2012DS. Almost all these QTLs are highly season-specific; only one locus (RM582 on chromosome 1) was consistently identified in both the 2011DS and combined analysis. These yield QTLs were on chromosomes 1, 2, 3, 5, 6, 7, 8, 10 and 12. The PV of the yield QTLs varied from 12% to 27%. Five QTLs had more than 20% PV. For DTF, 16 and 14 QTLs were identified under drought stress and NS, respectively; whereas, for PH, 13 and 17 QTLs were identified under drought stress and NS, respectively, and the results are provided in Additional file 3: Tables S4 and S5.

**Table 2:** Correlation among yield and yield-related traits

Trait/Season	Environment	PH	GY
DTF			
2011DS	S	0.256**	−0.025
2011DS	NS	0.051	−0.174
2012DS	S	−0.246**	−0.494**
2012DS	NS	0.449**	0.063
Combined	S	−0.175	−0.456**
Combined	NS	0.404**	0.023
PH		–	
2011DS	S	–	−0.152
2011DS	NS	–	−0.009
2012DS	S	–	−0.144
2012DS	NS	–	0.195*
Combined	S	–	0.327**
Combined	NS	–	0.310**

** significant at 1%, * significant at 5%

**Table 3:** Markers significantly associated with grain yield under drought stress

Marker	Chr	F-Marker	*p-*Marker	*R* ^*2*^	Year/Season
RM262	2	2.4772	0.0258	7.6	combined
RM26	5	4.4261	0.0031	7.4	combined
		5.3105	8.93E–04	19.1	11DS
		2.9933	0.0246	7.8	12DS
RM249	6	2.4746	0.0259	11.1	12DS
RM25694	10	2.8118	0.0231	13.9	11DS
RM224	11	2.4361	0.0436	5.6	combined
		3.2531	0.011	10.3	12DS
RM552	11	3.6227	0.0173	4.7	combined
		3.9686	0.0115	7.7	12DS
RM28048	12	3.8536	0.0071	14.8	11DS
		2.6494	0.0407	7.0	12DS

**Table 4:** Markers significantly associated with grain yield under non-stress

Marker	Chr	F-marker	*p-*value	*R* ^*2*^	Year/Season
RM11943	1	3.494	0.0202	12.6	11DS
RM582	1	2.805	0.0101	24.8	11DS
	1	2.255	0.0346	22.1	combined
RM12979	2	2.492	0.0312	17.7	11DS
RM521	2	3.373	0.0233	12.2	11DS
RM555	2	2.563	0.0352	16.2	combined
RM15983	3	2.817	0.0229	16.6	11DS
RM334	5	2.139	0.0391	23.6	combined
RM19637	6	2.876	0.0293	13.8	11DS
RM248	7	2.237	0.0269	26.7	combined
RM408	8	2.821	0.0317	13.6	11DS
RM24932	10	3.442	0.0052	24.0	combined
RM304	10	2.887	0.0148	11.6	12DS
RM491	12	1.927	0.0491	16.1	12DS

### Genes Within *qDTYs* Regions

All the *qDTYs* identified by both GLM and MLM during both the 2011DS and 2012DS were mapped into the physical genome and binned as meta-QTLs (described in the data of the methods section), resulting in 20 QTL bins or meta-QTLs representing 18.1 MB of the reference genome (Additional file 4: Table S6). The physical reference genome of variety Nipponbare was used to survey the genes in the meta-QTLs (IRGSP 1.0; Kawahara et al. 2013). We restricted the gene survey to a 1 MB region, covering 500 Kb regions toward the left and right side of the peak SSR marker to survey the genes more precisely. Using the Rice Genome Annotation Project (RGAP release 7, http://rice.plantbiology.msu.edu/), information on genes within the meta-QTL regions was gathered. In all, 3222 genes were found within the entire 20 meta-QTL regions, out of which 22% were transposable elements, 27% were uncharacterized expressed proteins and hypothetical proteins, and the remaining 53% were unique genes/gene families with assigned definitive functions (Additional file 5: Table S7). In all, there were 541 unique genes/gene families within the meta-QTLs and predominantly their putative functions were related to stress tolerance, stress signaling, hormonal regulation, growth and development, nutrient uptake, metal carriers and transcription factors. Some of the important genes/gene families with a high frequency of presence in the meta-QTLs were F-box domain proteins, MADS-box proteins, tetra- and pentatricopeptide proteins, no apical meristem proteins (NAM), heat shock proteins (HSPs), cytochrome P450 family, ethylene response factors (AP2/ERFs), auxin response factors (ARFs), brassinosteroids, CaMK, GDSL-like lipases, membrane proteins, hydrolases, kinases, peroxidases, peptidase and dismutase family proteins, zinc finger proteins (ZFPs), leucine zipper motifs (ZIP) and myeloblastosis (MYB) transcription factors. The role of these genes/gene families in abiotic stress tolerance is well known. Enrichment analysis of the list of genes within meta-QTLs against annotation of the RGAP genes for gene ontology (GO) identified a wide range of complex processes, functions and compartments/major sites of biochemical functions involved in abiotic stress tolerance. In all, 63 GO processes, 70 GO functions and 23 compartments related to stress tolerance, stress signaling, growth and development, and key metabolic processes were identified. MapMan pathway analysis for the genes located within the meta-QTLs identified 90 key biochemical pathways that might be conferring drought tolerance. QTARO of the meta-QTLs resulted in 160 curated QTLs significantly enriched in the gene lists (http://qtaro.abr.affrc.go.jp/qtab/table.). More than 60 (38%) curated QTLs were related to abiotic stress tolerance; others were related to growth and development, grain quality traits and disease resistance. There are differences in specific biological themes that are enriched per QTL bin but, in general, most of them had relevance to abiotic stress tolerance (Additional file 5: Table S7).

## Discussion

The adverse effects of climate change such as drought and heat are becoming a major threat to sustainable rice production and productivity (IPCC et al., 2007). In recent years, El Niño-induced drought has become a common phenomenon across many countries of Asia and there is already a clear prediction that El Niño-induced drought will have a significant effect on overall rice stocks in 2016 (Mohanty, 2015). In order to mitigate drought, there have been increased efforts to breed drought-tolerant rice varieties by both conventional and molecular breeding approaches (Kumar et al. 2014). Several major-effect QTLs for GY under drought have been identified for both upland and lowland conditions (Kumar et al. 2014). These QTLs have been successfully used in marker-assisted backcross (MABC) breeding programs to improve the drought tolerance of widely adopted popular but drought-susceptible rice varieties such as IR64, Swarna, MR219 and MRQ74 (Swamy et al. 2013; Swamy et al. 2014; Noraziyah et al. 2016a; Noraziyah et al. 2016b). However, *qDTYs* have different synergistic effects in different genetic backgrounds and meta-analysis of the major *qDTYs* has shown that few hotspot QTL regions are repeatedly being identified. To gain an economic yield advantage of at least 500 kg ha^−1^, a minimum of two to three *qDTYs* have to be pyramided in elite genetic backgrounds (Swamy and Kumar, 2012); thus, exploration of genetic resources and the identification of several new *qDTYs* by association mapping is one of the most promising approaches.

Association analysis using germplasm collections has been successfully used to identify several major loci for different traits (Han and Huang 2013; Wu et al. 2015; Yang et al. 2014), but only a few association studies exist for drought-related traits in rice (Vasant, 2012; Courtois et al. 2013; Vannirajan et al. 2012; Muthukumar et al. 2015). There is thus large scope to explore germplasm through association mapping to identify loci linked to drought tolerance.

### Phenotypic Analysis

Drought stress was successfully created in the trials with controlled irrigation and the screening was supported by less rainfall during the cropping seasons, which resulted in a drastic reduction in the water table. The lesser amount of rainfall and longer dry spells are the key components for successful drought screening at IRRI during the dry season (Vikram et al. 2011). Some 68.8% of the accessions reached 50% flowering by the end of March. The remaining germplasm has early and late flowering time, by 5.1% and 21.6%, respectively. This indicates that the crops received severe stress for about two weeks during panicle development to pre-flowering stage. Jennings et al. (1979) reported that exposure to at least two weeks of drought stress due to rainless days during the vegetative stage and at least one week during the reproductive stage can differentiate susceptible and drought-tolerant genotypes.

The delayed flowering observed under stress in the present study is in agreement with earlier reports (Vikram et al. 2011; Jongdee et al. 2006; Zhao et al. 2010; Atlin et al. 2006; Lafitte and Courtois, 2002; Pantuwan et al. 2002b). According to Pantuwan et al. (2002b), delayed flowering under drought stress could be a good measurement of plant responses and adaptability to drought tolerance, and also an efficient selection criterion for distinguishing drought-susceptible and drought-tolerant genotypes. The negative effect of drought on performance in many traits, including yield, has been reported. Even mild stresses during flowering cause a severe reduction in PH, biomass, spikelet fertility and GY. The reduction is mainly because of water shortage in the plant causing more respiration and reduced photosynthesis, leading to less biomass accumulation and less GY (Fukai et al. 1999; O’Toole, 1982; Boonjung and Fukai, 1996).

The yield reduction in the trials varied from 51% to 60%, indicating that the association panel was subjected to moderate to severe drought stress. A yield reduction of more than 50% was also reported in earlier studies, indicating successful drought screening (Venuprasad et al. 2009; Vikram et al. 2011; Ghimire et al. 2012; Noraziyah et al. 2016a; Noraziyah et al. 2016b). Poor panicle exsertion, reduced number of spikelets and poor grain filling are some of the causes of reduced GY under drought stress (Pantuwan et al. 2002a; O’Toole and Namuco, 1983; Cruz and O’Toole, 1984; Ekanayake et al. 1989). Our results once again reaffirm the use of GY under drought stress as an effective criterion for improving drought tolerance in rice. The CV and ranges for various traits clearly indicated the wide variations for yield and yield-related traits under both drought stress and NS, so there are some good drought-tolerant donor lines in the panel that can be used in drought breeding programs by combining both drought tolerance and high yield potential.

The H^2^ for GY under drought stress was more than 70%, indicating that selection for GY under drought stress may be effective for improving drought tolerance. Moderate to high H^2^ of GY under drought stress conditions was also observed in many studies (Babu et al. 2003; Atlin et al. 2004; Lafitte et al. 2004; Kumar et al. 2008; Kamoshita et al. 2008). High H^2^ in PH and DTF under drought stress was also observed in several studies (Lafitte et al. 2004; Lanceras et al. 2004; Bernier et al. 2007). Gomez et al. (2006) concluded that the high H^2^ in these traits was due to the preponderance of additive gene action and was suitable for direct selection in improving drought tolerance in rice. In the present study, the moderate to high H^2^ values for yield and yield-related traits indicate that they are genetically controlled by additive gene action and can be used as a selection parameter under both drought stress and non-stress environments. This indicates that the genotypes that have early flowering time may have a smaller yield reduction. This negative correlation was also observed in earlier studies (Vikram et al. 2011, 2016; Zhao et al. 2010). The genotypes with early flowering time have higher GY than the genotypes that flowered later (Pantuwan et al. 2002b).

### Genotypic Analysis

There is a wider molecular diversity within the association panel (almost 95% of the SSR markers tested were polymorphic) and in total 119 SSR markers amplified 1524 alleles in the 75 genotypes. The number of alleles per loci varied from 2 to 13, with an average of 8.36 per loci. The amount of diversity available within this association panel is comparable with or even higher than that of earlier reports (Agrama et al. 2007; Yan et al. 2009; Borba et al. 2010; Muthukumar et al. 2015; Raju et al. 2016). In general, higher genetic diversity in any germplasm collection indicates lesser gene flow among the accessions (Rahman et al. 2007; Dinesh Raj et al. 2010). This was also true in our panel as the genetic diversity was very high, perhaps because the germplasm consisted of landraces, breeding lines and introductions with various origins and collected from different geographic regions of Malaysia.

One of the main concerns of association mapping is the spurious marker trait associations due to population structure (Krill et al. 2010). In association mapping studies, it has been a common practice to perform structure analysis in any structured populations to overcome spurious associations (Pritchard et al. 2000). All the 75 genotypes used in our study were from the *indica* subgroup but the structure analysis grouped them into three subgroups (K = 3) with admixtures. Nearly 40 (53%) of the genotypes were clearly grouped into one of the subgroups, while 35 (47%) genotypes showed some amount of allelic reshuffling or sharing among different subgroups. Such allelic sharing among different genotypes has been attributed to the accumulation of several spontaneous mutations among genotypes from different geographic areas (Mather et al. 2004; Agrama et al. 2007).

The molecular diversity-based clustering also grouped the genotypes into three clusters, confirming the results of the structure analysis indicating the clear divergence among three subgroups (Fig. 1). This kind of clear differentiation among genotypes of any subspecies may be because of their different growth environments, adaptive traits and evolutionary patterns. The allelic fixation (F_st_) values were different among the three subgroups and varied from 0.194 to 0.277, suggesting higher genome compatibility among the genotypes, indicating that crosses made between the genotypes from these distinct subgroups would generate superior lines with better adaptability to moderate to severe drought conditions (N’Goran et al. 2000; Raju et al. 2016). The expected heterozygosity was higher in all the three subgroups and it was more than 0.544 among the three subgroups.

LD analysis was carried out for the all the marker pairs and 32% of the pairs were significant (*P* > 0.05). The number of significant intra- and inter-chromosomal LD pairs varied on different chromosomes. Chromosome 2 had the highest number of significant intra- and inter-chromosomal LD pairs, whereas chromosome 7 had the lowest number. There was a reduction in number of significant LD pairs as the interval distances between marker pairs increased. The LD plotted against r^2^ and LD plotted against cM is presented in Fig. 2. There was a sharp decline in LD decay for the linked markers at 5-20 cM. Overall, the r^2^ varied from 0.005 to 0.31, with an average of 0.03. Significant pairs of linked loci (r^2^ > 0.2) showed an average distance of 25 cM, indicating the presence of a higher number of significant LD blocks in this set of genotypes. The larger LD blocks within a germplasm collection are highly useful in genome-wide association mapping. There are numerous reports of LD patterns in rice. Olsen et al. (2006) and Mather et al. (2007) reported LD decay occurring at about 1 cM distance, whereas others reported LD decay at 20–30 cM distances using SSR markers (Agrama et al. 2007; Agrama and Eizenga, 2008; Jin et al. 2010; Vannirajan et al. 2012). Several factors such as nature of pollination, geographic isolation, evolutionary pattern, mutation, selection pressure and genetic drift influence the size and number of LD blocks (Gupta et al. 2005). In a predominantly self-pollinated crop species such as rice, larger LD blocks extending over several cM are usually expected (Abdurakhmonov and Abdukarimov, 2008); also, LD varied among different subspecies of rice. The extent of LD in the *indica* subspecies was lesser than in *temperate japonica* or *tropical japonica* (Khush, 1997; Garris et al. 2005; Mather et al. 2007).

### Association Analysis

The structured association mapping of yield and yield-related traits revealed that there were 198 and 80 significant marker trait associations (MTA) for all three traits by GLM and MLM analysis and most of these loci were similar to the major loci reported for these traits (Bernier et al. 2007; Venuprasad et al. 2009; Swamy et al. 2011; Vikram et al. 2011, 2015; Ghimire et al. 2012; Palanog et al. 2014; Courtois et al. 2013; Kumar et al. 2014). In the genotypic analysis, seven out of the 119 SSR markers used were linked to known major-effect drought GY QTLs identified at IRRI (Kumar et al. 2014). Thus confirming the consistency, accuracy and effectiveness of these QTLs, and also most of them were in the drought QTL hotspot regions (Swamy et al. 2013). The existence of such large-effect and consistent QTLs is one of the main reasons for successful MAB for improving the drought tolerance of several popular but drought-susceptible rice varieties (Noraziyah et al. 2016a; Noraziyah et al. 2016b). It is also interesting to note that major-effect QTLs *qDTY*_*12.1*_, was present in 10.6% of the genotypes, and a similar result was also reported by Swamy et al. (2011). In addition, we have identified a few new major loci for GY under drought stress on chromosomes 5, 6 and 11 with a PV of 5–19%. It is interesting to that the novel QTL on chromosome 5 was consistently identified in all the three analyses and had highest PV of 19%. These QTLs are useful for MAB in combination with other major-effect *qDTYs*. Eventhough some major effect QTLs were identified for three traits, the results could have been improved by increasing the marker density and the population size. Several consistent major-effect QTLs were also identified for GY under non-stress, but most of them were season-specific except for one locus (RM582) on chromosome 7. The overall results have clearly shown that association mapping is one of the feasible options for identifying major-effect QTLs for GY under drought.

The low marker density of one in every 3 MB and a smaller population size of 75 lines used in this study might have impacted the identification of new QTLs. Recently genome wide association analysis (GWAS) for complex traits using high density single nucleotide polymorphism markers (SNP) are becoming common, it provides high mapping resolution and high accuracy of the QTLs due to better structuring of the populations (Han and Huang, 2013; Wu et al. 2015; Yang et al. 2014). Therefore, we suggest the QTLs identified in the present study may be further reconfirmed by increasing marker density and size of the association panel.

### Enrichment Analysis of Genes Within *qDTYs*

The binning of all the *qDTYs* resulted in 20 meta-QTLs spanning 18 MB, which is 4.6% of the reference genome; this clearly shows that binning has been successful in reducing the QTL regions without losing significant information on gene content. The binned multiple QTL regions with less than 10% of the genome size are normally considered useful in identifying the genes/gene families underlying target traits. The materials used in this study are from diverse sources and there is no physical map that exactly matches the genome of these materials. However, there are more than 98% similarities among the genomes of different rice varieties and the available reference genome information can be used in gene assessment in a wide range of germplasm (Schatz et al. 2014). We used the Nipponbare physical map as a reference genome for gene survey, identification of processes, pathways and curation of the QTLs. Enrichment analysis of the genes within QTLs against the genome-wide annotation for functions/processes/cell compartments (GO), pathways (MapMan) and association to previously published QTLs (QTARO) shows significantly enriched (P < 0.005) interesting biological processes that may be working toward achieving improved yield under drought (e.g., GO:0005975: carbohydrate metabolic process; GO:0006950: response to stress; MapMan 20.1.5: stress. biotic.regulation of transcription) as well as significant association of the discovered QTLs to previously mapped QTLs for drought tolerance (e.g., 21 drought tolerance genes affecting spikelet fertility, root volume/biomass/length, leaf rolling, stomatal resistance and various yield-correlated traits, in supplemental file < DEF_enrichment_QTL-genes_p005.xlsx>) in the QTARO database. This additional information about these contiguous genes offers further inferences on how a QTL functions to deliver the trait expected, and helps to guide in the selection of which QTLs could be more effective in a breeding program.

Here, we provide glimpses of the important genes/gene families associated with the drought QTL regions and evidence of their role in drought tolerance is discussed. The prominent genes/gene families enriched within the drought QTLs were NAC transcription factors, F-box domain proteins, MADS-box proteins, tetra- and pentatricopeptide proteins, NAC, NAM, HSPs, cytochrome P450 family, AP2/ERFs, ARFs, brassinosteroids, CaAMK, GDSL-like lipases, membrane proteins, hydrolases, kinases, peroxidases, peptidase and dismutase family proteins, ANC, ZFPs, ZIP, and MYB transcription factors (TFs). The role of these genes/gene families in drought stress tolerance has been reported in rice and other crops.

Root traits play an important role in enhancing drought tolerance in rice. Several TFs such as ARF, MYB, ZIP, NAC, NAM and MADS-box genes were involved in changes in root architecture and they help in tolerating drought stress (Okushima et al. 2007; Xie et al. 2000; Zhang and Forde, 1998). *OsNAC1* has been reported to increase the number of lateral roots under drought stress, while *OsNAC10* resulted in thicker roots (Khong et al. 2008; Jeong, 2010). Since grain yield under drought is the most economic component under drought, floral meristic tissue activities, floral organ development, control of flowering time and pollen function play an important role in spikelet development and filling of spikelets to produce grains under drought stress (Hepworth et al. 2006; Imaizumi et al. 2003; Sonneveld et al. 2005). Genes such as *OsGILP*, ZIP, cytochrome P450 family and F-box family genes are involved in floral development and grain production under stress conditions, including drought. Heat shock family proteins improve heat tolerance by improving plant physiological phenomena such as photosynthesis; assimilate partitioning, water and nutrient use efficiency, and membrane stability (Wahid et al. 2007). ERF/AP2 domain proteins such as *OsERF1*, *OsERF2* and *OsDREBs* enhance the osmotic and drought tolerance of rice by modulating the increase in stress-responsive gene expression through different pathways (Dubouzet et al. 2003; Gao et al. 2008; Chen et al. 2008; Mishra et al. 2009; Quan et al. 2010; Fukao and Serres, 2008). The over expression of *OsbZIPs* has increased drought stress tolerance in rice, whereas its down-regulation/knockout leads to higher sensitivity (Xiang et al. 2008; Lu et al. 2009). Zhang et al. (2016) reported that the ZFP and MYB protein families also play an important role in response to water stress, such as the regulation of stomatal movement, synthesis of suberin and cuticular wax and the regulation of flower development (Xiong et al. 2014; Ma et al. 2009; Baldoni et al. 2015). All these evidences clearly indicate that drought QTLs were enriched with several genes/gene families involved in drought stress responses and their coordinated action is essential for conferring drought tolerance and producing yield under drought stress. However, we suggest further in-depth analysis of the entire drought yield QTLs reported to gain more insight into the genes underlying drought QTLs and their molecular mechanisms involved in conferring drought tolerance.

## Conclusions

Drought is a severe abiotic stress affecting rice production. The identification and introgression of major-effect QTLs are one of the best and proven approaches to improving the drought tolerance of rice varieties. The accuracy and consistency of QTLs have clearly shown that structured association mapping with genome-wide molecular markers is an attractive option to identify major-effect QTLs for GY under drought stress. Some of the new *qDTYs* identified in this study are useful for MAB in combination with other major *qDTYs.* The *in silico* analysis of QTL regions revealed that several drought-responsive genes were associated with the grain yield under drought.

## Methods

### Plants Materials

We used 75 rice genotypes in this study. The seeds were collected from the Rice Gene Bank of MARDI, Seberang Perai, Malaysia. The material consisted of Malaysian landraces, breeding lines, varieties, cultivars and introductions (Additional file 3: Table S8). Six drought-tolerant checks (Vandana, Apo, PSBRC-82, UPLRi7, Mokwoo and IR77298-14-1-2-10) and two drought-susceptible checks (IR64 and MTU1010) were included in the experiment.

### Genotyping

Molecular work was conducted in the Genotyping Services Laboratory (GSL) of the Plant Breeding Division at IRRI.

#### DNA Extraction and PCR Amplification

Fresh leaf samples were collected from each accession three weeks after transplanting and were freeze-dried using a lyophilizer. The DNA was extracted by using a modified CTAB protocol. This version of DNA extraction method was developed by Murray and Thompson (1980); it uses cetyl trimethyl ammonium bromide (CTAB). Agarose gel electrophoresis was used to check the quality and quantity of DNA extracted. The concentration of the isolated DNA was estimated based on the band brightness and thickness compared with those of the reference λ DNA. The DNA samples were diluted with 1x TE into an equal concentration of 25 ng/ul.

Amplification of SSR markers using polymerase chain reaction (PCR) was done with a 15 μl reaction mixture that contained 3 μl of DNA template, 1.5 μl of 10x PCR buffer, 2.0 μl of MgCl_2_, 0.5 μl each of forward and reverse primers, 0.5 μl of 1 mM dNTP and 0.5 μl of Taq DNA polymerase (1:20 homemade). A drop of mineral oil was added on each well to prevent the mixture from evaporating and the plate was covered with PCR sealing film. Finally, 10x loading buffer was added to the PCR product prior to loading (1.3 μl of 10x loading buffer for every 10 μl of PCR product). Amplification reaction was carried out in a 96-well PCR plate in a thermocyler. The following PCR profile was used for SSR amplification: initial denaturation at 94 °C for 5 min and then 35 cycles of denaturation at 94 °C for 30 s, annealing at 55 °C for 30 s and extension at 72 °C for 30 s; and final extension at 72 °C for 5 min and storage at 10 °C forever. The PCR products were resolved using high-resolution 8% polyacrylamide gel electrophoresis (PAGE) as described by Sambrook and Russell (2001). The gel was run in 1x TBE at 95 volts for 1 to 3 hours depending on the product size of the SSR marker. Gels were stained with SYBR Safe^TM^ DNA gel stain and were viewed after 20 minutes.

#### Marker Analysis

We used 119 highly polymorphic SSR markers for genotyping all 75 genotypes. Out of 119 markers, 45 markers were linked to major-effect grain yield QTLs under drought such as *qDTY*_*1.1*_ *, qDTY*_*2.1*_ *, qDTY*_*2.2*_ *, qDTY*_*3.1*_ *, qDTY*_*4.1*_ *, qDTY*_*9.1*_ *, qDTY*_*10.1*_ *, qDTY*_*12.1*_ *, qDTY*_*1.2*_ and *qDTY*_*2.3*_ and all the remaining markers were randomly distributed within the genome (Additional file 3: Table S9). The details of marker information such as chromosome number, position (cM), expected product size and annealing temperature were obtained from the Gramene database (http://www.gramene.org).

##### Phenotyping Under Reproductive Stage (RS) Drought Stress and Non-stress (NS)

The phenotyping was conducted at the International Rice Research Institute (IRRI), Los Baños, Laguna, Philippines, during the 2011DS and 2012DS. IRRI is located at 14^o^13‘N latitude, 121^o^15‘E longitude, at an altitude of 21 m above mean sea level. The soil type of the experimental field is a Mahaas clay loam, isohyperthermic mixed typic tropudalf. RS and NS trials were established in an alpha lattice design with two replications in two-row plots with 2 m in length and plant spacing of 20 cm between rows and 20 cm between plants with standard agronomic practices. Both RS and NS trials were planted in the 2011DS and repeated in the 2012 DS. The NS trial was managed like irrigated lowland where no water stress was employed. The purpose of the NS trial was to serve as a source of seed for the succeeding trial. Conversely, the RS trial refers to a trial in which drought stress is artificially imposed during the reproductive stage. In the RS trial, water was drained in the field at four weeks after transplanting to start the imposition of drought stress. Perforated PVC pipes were strategically installed in six locations in the field to monitor for parching water table daily. The parching water table was measured in all pipes until the crops reached 50% maturity. When the water table reached 100 cm below the soil surface for about three weeks and 70% of the plants showed severe leaf rolling and seemed beyond recovery, irrigation was immediately applied through flooding for 6 hours and the water was drained again after the field was saturated.

After transplanting, 2–5 cm of standing water was maintained in the field. Drought was imposed at 30 days after transplanting (DAT) by draining the water in the field and withholding it until soil moisture tension reached -70 kPa at 0.2-m depth. The fields were re-irrigated by flooding for 24 hours. This was repeated during harvesting. The soil moisture level below the soil surface was regularly monitored and measured through the observation wells strategically installed in the field. Fertilizer was applied three times at 10, 25 and 45 DAT at the rate of 90-30-30 kg NPK ha^−1^.

##### Data recording and analysis

Observations were recorded at different stages of crop growth until maturity from both RS and NS trials. Phenotypic data recorded were days to 50% flowering (DTF), plant height (PH) and grain yield (GY). The phenotypic observations were analyzed using PB Tools to estimate the trial mean, range, SD, CV, broad-sense heritability (H^2^) and genetic advance (GA). Correlations among the traits under drought stress and NS were estimated using STAR (v17.0).

### Association Analysis

#### Allelic Diversity and Cluster Analysis

Allele frequency, genetic diversity, polymorphic information content (PIC) and molecular diversity were assessed using PowerMarker (v3.25). The linkage disequilibrium between marker pairs was tested at 1% significance level by PowerMarker (v3.25).

#### Population Structure

Based on the genotyping data from 119 SSR markers that were evenly distributed across all 12 chromosomes, population structure was estimated with the model-based (Bayesian) cluster software STRUCTURE 2.2 (Pritchard et al. 2000). The software was set to have length of burn-in period of 500,000 followed by 500,000 Markov chain Monte Carlo (MCMC) reps after burn-in. The optimum number of populations was inferred by running an admixture ancestry model with correlated frequencies starting from two populations, *K* = 2 to *K* = 10, with three replications at each *K*. The optimum value of *K =* 3 was determined, thus indicating that all the germplasm could be divided into three subgroups: SG1, SG2 and SG3.

#### Association Mapping

Association between markers and three traits (DTF, PH and GY) under RS drought stress and NS was calculated using TASSEL2.1. We used generalized linear model (GLM) and Mixed Linear Model (MLM) functions for the analysis. The marker and trait association was declared significant when *P* < 0.05.

#### In Silico *Analysis of qDTY Regions*

All the SSRs reported to be associated with a *qDTY* as a union set (all markers in two-season trials as one set) were mapped to the IRGSP 1.0 genome by aligning the SSR primer sequences using BLAT (Kent, 2002) with parameter tile size 11 for maximum sensitivity. Since this is a single-marker regression QTL mapping, we estimated the physical interval size of a QTL based on most of the original drought QTLs to be around a 1-MB region. Regions defined by a single SSR marker were extended to include 500 kb left and 500 kb right of each individual marker. We call these QTL genome intervals associated bins (they are not true QTLs), and they represent +/−0.5 mb to the left and right of the region flanked by the SSR forward and reverse primer, except for three bins that were merged from two adjacent SSR regions, which may be slightly bigger than 1 mb or as small as 20 kb only (Additional file 4: Table S6). Twenty associated bins that represent 18 megabases of the reference genome were determined and 3222 RGAP genes were located within these associated bins, which were used for the enrichment analyses against the annotation of all the RGAP genes with gene ontology (GO) terms (RGAP release 7), MapMan pathway annotation (MapMan URL) and QTARO QTL association (http://qtaro.abr.affrc.go.jp/).

## Additional files

Additional file 1: Figure S1. Daily rainfall during the dry season experiment period from February to May in 2011. (DOCX 12 kb)

Additional file 2: Figure S2. Daily rainfall during the dry season experiment period from February to May in 2012. (DOCX 12 kb)

Additional file 3: **Table S1.** Various population parameters measured in the germplasm. **Table S2.** Subpopulation-specific statistical parameters. **Table S3.** Number of intra- and inter-chromosomal linkage disequilibrium (LD) pairs. **Table S4.** Markers significantly associated with DTF and PH under drought stress. **Tables S5.** Markers significantly associated with DTF and PH under non stress. **Table S8.** List of *Oryza* accession used in this study assemble in their origin. **Table S9.** List of random and specific microsatellite markers group according to the chromosome number. (DOCX 47 kb)

Additional file 4: Table S6. List of genes in association bins for grain yield under drought. (XLSX 141 kb)

Additional file 5: Table S7. Enrichment analysis of drought resposive genes in the drough QTLs. (XLSX 265 kb)

## Abbreviations

CV: Coefficient of variation
DS: Dry season
DTF: Days to 50% flowering
GSL: Genotyping Services Laboratory
GY: Grain yield
IRRI: International Rice Research Institute
LD: Linkage disequilibrium
NS: Non-stress
PH: Plant height
POP: Population
*qDTY*: Drought yield QTL
