# Supplementary material for: Association Mapping of Yield and Yield-related Traits Under Reproductive Stage Drought Stress in Rice (Oryza sativa L.)
Source: Rice (N Y). 2017 May 18;10:21. doi: 10.1186/s12284-017-0161-6 (PMC5436998; doi:10.1186/s12284-017-0161-6)
Supplement: Supplementary file 2 — Daily rainfall during the dry season experiment period from February to May in 2012. (DOCX 12 kb) [file 12284_2017_161_MOESM2_ESM.docx]

**Fig S2 Daily rainfall during the dry season experiment period from February to May in 2012**
