# Supplementary material for: Association Mapping of Yield and Yield-related Traits Under Reproductive Stage Drought Stress in Rice (Oryza sativa L.)
Source: Rice (N Y). 2017 May 18;10:21. doi: 10.1186/s12284-017-0161-6 (PMC5436998; doi:10.1186/s12284-017-0161-6)
Supplement: Supplementary file 3 — Table S1. Various population parameters measured in the germplasm. Table S2. Subpopulation-specific statistical parameters. Table S3. Number of intra- and inter-chromosomal linkage disequilibrium (LD) pairs. Table S4. Markers significantly associated with DTF and PH under drought stress. Tables S5. Markers significantly associated with DTF and PH under non stress. Table S8. List of Oryza accession used in this study assemble in their origin. Table S9. List of random and specific microsatellite markers group according to the chromosome number. (DOCX 47 kb) [file 12284_2017_161_MOESM3_ESM.docx]

**Table S1. Various population parameters measured in the germplasm**

| **Chromosome** | **Allele**  **frequency** | **Alleles** | **Diversity** | **PIC** | **Number of population-specific alleles** | | |
| --- | --- | --- | --- | --- | --- | --- | --- |
|  |  |  |  |  | **POP1** | **POP2** | **POP3** |
| Chr1 | 0.460 | 6.235 | 0.673 | 0.634 | 7 | 9 | 10 |
| Chr2 | 0.440 | 6.250 | 0.679 | 0.637 | 3 | 9 | 8 |
| Chr3 | 0.467 | 5.875 | 0.656 | 0.610 | - | 3 | 3 |
| Chr4 | 0.440 | 7.143 | 0.690 | 0.654 | 4 | 3 | 2 |
| Chr5 | 0.382 | 8.000 | 0.760 | 0.729 | 4 | 6 | 3 |
| Chr6 | 0.457 | 6.857 | 0.699 | 0.660 | 1 | 7 | 3 |
| Chr7 | 0.594 | 6.429 | 0.569 | 0.536 | 1 | 4 | 6 |
| Chr8 | 0.467 | 5.667 | 0.647 | 0.590 | 1 | 4 | - |
| Chr9 | 0.405 | 6.455 | 0.708 | 0.670 | 4 | 3 | 7 |
| Chr10 | 0.437 | 6.100 | 0.663 | 0.627 | 2 | 3 | 2 |
| Chr11 | 0.411 | 7.429 | 0.704 | 0.669 | 2 | 4 | 4 |
| Chr12 | 0.528 | 5.462 | 0.586 | 0.544 | - | 2 | 13 |
| Total | 0.458 | 6.370 | 0.667 | 0.627 | 29 | 57 | 61 |

**Table S2. Subpopulation-specific statistical parameters**

| **Population** | **Membership (%)** | **FST** | **Expected heterozygosity** | **Allelic frequency divergence** | | |
| --- | --- | --- | --- | --- | --- | --- |
|  |  |  |  | **POP1** | **POP2** | **POP3** |
| POP1 | 30.6 | 0.277 | 0.544 | - | - | - |
| POP2 | 24.0 | 0.270 | 0.572 | 0.194 | - | - |
| POP3 | 45.4 | 0.194 | 0.559 | 0.120 | 0.197 | - |

**Table S3. Number of intra- and inter-chromosomal linkage disequilibrium (LD) pairs**

| **Chromosome** | **Intra-chromosomal** | | **Inter-chromosomal** | | **Total** | |
| --- | --- | --- | --- | --- | --- | --- |
|  | **LD pairs** | **P-value** | **LD pairs** | **P-value** | **LD pairs** | **P-value** |
| 1 | 30 | <0.05 | 122 | <0.05 | 152 | <0.05 |
| 2 | 67 | <0.01 | 370 | <0.05 | 437 | <0.05 |
| 3 | 13 | <0.05 | 182 | <0.05 | 195 | <0.05 |
| 4 | 3 | <0.05 | 127 | <0.05 | 130 | <0.05 |
| 5 | 6 | <0.05 | 138 | <0.04 | 144 | <0.05 |
| 6 | 12 | <0.04 | 213 | <0.05 | 225 | <0.05 |
| 7 | 1 | <0.03 | 83 | <0.05 | 84 | <0.05 |
| 8 | 6 | <0.05 | 81 | <0.04 | 87 | <0.05 |
| 9 | 21 | <0.05 | 213 | <0.05 | 234 | <0.05 |
| 10 | 21 | <0.05 | 212 | <0.04 | 233 | <0.05 |
| 11 | 8 | <0.05 | 115 | <0.05 | 123 | <0.05 |
| 12 | 5 | <0.05 | 215 | <0.02 | 220 | <0.05 |
| Overall | 193 | <0.05 | 2071 | <0.05 | 2264 | <0.05 |

**Table S4 Markers significantly associated with days to flowering under drought stress**

| **Trait** | **Marker** | **Chr** | **F-Marker** | **p-value** | **R^2^** | **Year/Season** |
| --- | --- | --- | --- | --- | --- | --- |
| DTF | RM10488 | 1 | 2.2812 | 0.0387 | 16.0 | combined |
| DTF | RM12091 | 1 | 2.257 | 0.0345 | 17.9 | combined |
| DTF | RM12233 | 1 | 3.0264 | 0.0235 | 12.3 | combined |
| DTF | RM522 | 1 | 2.7451 | 0.0497 | 8.8 | combined |
| DTF | OSR17 | 2 | 2.2566 | 0.0407 | 19.7 | 11DS |
| DTF | RM521 | 2 | 3.0033 | 0.0364 | 11.6 | 11DS |
| DTF | RM555 | 2 | 3.5175 | 0.007 | 20.9 | 11DS |
| DTF | RM17524 | 4 | 2.5943 | 0.0202 | 22.1 | 12DS |
| DTF | RM255 | 4 | 2.7436 | 0.0355 | 14.0 | 11DS |
| DTF | RM26 | 5 | 2.5647 | 0.0461 | 10.8 | combined |
| DTF | RM20776 | 7 | 3.355 | 0.0145 | 16.6 | 11DS |
|  |  | 7 | 3.5033 | 0.0117 | 17.3 | 12DS |
| DTF | RM21388 | 7 | 4.1716 | 0.009 | 15.5 | 12DS |
| DTF | RM223 | 8 | 6.6904 | 1.51E-05 | 38.1 | 12DS |
| DTF | RM24350 | 9 | 2.8839 | 0.0289 | 12.0 | combined |
|  |  | 9 | 2.7844 | 0.0334 | 14.2 | 12DS |
| DTF | RM296 | 9 | 2.4573 | 0.0269 | 17.1 | combined |
| DTF | RM28048 | 12 | 6.8953 | 1.04E-04 | 29.0 | 11DS |
| HT | RM582 | 1 | 3.3492 | 0.0029 | 14.1 | 12DS |
| HT | RM324 | 2 | 3.445 | 0.0024 | 14.4 | 12DS |
| HT | RM327 | 2 | 3.5805 | 0.0182 | 10.6 | 11DS |
| HT | RM521 | 2 | 2.693 | 0.0529 | 8.4 | 11DS |
| HT | RM6374 | 2 | 2.4263 | 0.0143 | 14.6 | 12DS |
| HT | RM17524 | 4 | 2.86 | 0.0116 | 18.9 | 11DS |
| HT | RM255 | 4 | 3.2123 | 0.0179 | 7.7 | 12DS |
| HT | RM249 | 6 | 2.6517 | 0.018 | 17.8 | 11DS |
| HT | RM400 | 6 | 2.8108 | 0.0099 | 12.4 | 12DS |
|  |  | 6 | 3.4206 | 6.09E-04 | 20.8 | 12DS |
| HT | RM180 | 7 | 2.2794 | 0.0242 | 12.9 | 12DS |
| HT | RM20776 | 7 | 4.13 | 0.0047 | 15.7 | 11DS |
|  |  | 7 | 4.9838 | 0.0014 | 11.0 | 12DS |
| HT | RM88 | 8 | 3.8973 | 0.0125 | 7.1 | 12DS |
| HT | RM28048 | 12 | 5.2897 | 9.19E-04 | 18.9 | 11DS |

**Tables S5. Markers significantly associated with plant height under drought non stress**

| **Trait** | **Marker** | **Chr** | **F-Marker** | **p-Marker** | **R^2^** | **Year/Season** |
| --- | --- | --- | --- | --- | --- | --- |
| DTF | RM10488 | 1 | 3.212 | 0.006 | 13.0 | 12DS |
| DTF | RM129 | 1 | 3.254 | 0.005 | 11.9 | combined |
|  |  | 1 | 2.692 | 0.017 | 11.6 | 12DS |
| DTF | RM3825 | 1 | 4.217 | 0.001 | 14.8 | combined |
| DTF | RM582 | 1 | 2.989 | 0.007 | 14.3 | 12DS |
| DTF | OSR17 | 2 | 2.700 | 0.016 | 11.5 | 12DS |
| DTF | RM12569 | 2 | 4.486 | 0.001 | 14.8 | 12DS |
| DTF | RM330 | 2 | 3.586 | 0.004 | 22.8 | 11DS |
| DTF | RM6374 | 2 | 3.372 | 0.001 | 18.2 | combined |
|  |  | 2 | 2.153 | 0.030 | 14.5 | 12DS |
| DTF | RM13 | 5 | 4.105 | 0.000 | 17.4 | combined |
| DTF | RM440 | 5 | 2.107 | 0.027 | 16.7 | 12DS |
| DTF | RM249 | 6 | 4.017 | 0.001 | 14.3 | combined |
|  |  | 6 | 2.526 | 0.023 | 11.1 | 12DS |
| DTF | RM219 | 9 | 2.445 | 0.023 | 21.8 | 11DS |
| DTF | RM444 | 9 | 2.284 | 0.046 | 16.0 | 11DS |
|  |  | 9 | 3.287 | 0.007 | 11.0 | combined |
| DTF | RM441 | 11 | 2.785 | 0.011 | 13.6 | 12DS |
| HT | RM10488 | 1 | 2.552 | 0.022 | 14.1 | 12DS |
| HT | RM28048 | 1 | 2.556 | 0.047 | 13.0 | combined |
| HT | RM28083 | 1 | 2.732 | 0.026 | 11.3 | 12DS |
| HT | RM582 | 1 | 2.355 | 0.028 | 15.5 | 12DS |
| HT | OSR17 | 2 | 2.378 | 0.032 | 13.3 | 12DS |
| HT | RM12569 | 2 | 3.932 | 0.002 | 17.4 | 12DS |
| HT | RM324 | 2 | 2.352 | 0.028 | 22.5 | combined |
| HT | RM521 | 2 | 4.261 | 0.008 | 15.5 | combined |
| HT | RM6374 | 2 | 2.396 | 0.016 | 29.9 | combined |
|  |  | 2 | 2.532 | 0.011 | 21.2 | 12DS |
| HT | RM13 | 5 | 2.464 | 0.018 | 25.8 | combined |
| HT | RM249 | 6 | 2.235 | 0.043 | 19.3 | combined |
| HT | RM469 | 6 | 2.383 | 0.048 | 15.0 | combined |
| HT | RM248 | 7 | 2.055 | 0.043 | 24.4 | 11DS |
| HT | RM444 | 9 | 2.250 | 0.049 | 16.9 | combined |
| HT | RM24932 | 10 | 4.905 | 0.000 | 30.2 | 11DS |
| HT | RM286 | 11 | 2.258 | 0.049 | 16.9 | combined |
| HT | RM441 | 11 | 2.794 | 0.010 | 17.6 | 12DS |

**Table S8. List of *Oryza* accession used in this study assemble in their origin**

| No | Accession Name |  |
| --- | --- | --- |
| 1 | MTU1010 |  |
| 2 | Vandana |  |
| 3 | 1989RP5-1 |  |
| 4 | 2060CR10-38-208 |  |
| 5 | Abong |  |
| 6 | Bangkok |  |
| 7 | BIRIS |  |
| 8 | CICA4 |  |
| 9 | CP231-H012 |  |
| 10 | Della |  |
| 11 | Huma Kuning Lenggong |  |
| 12 | Huma Wangi Lenggong |  |
| 13 | IET1991 |  |
| 14 | Jarom Mas |  |
| 15 | Jaya 3b-2 |  |
| 16 | Jayanti |  |
| 17 | Khao Lo |  |
| 18 | Kurau Wangi |  |
| 19 | Madu |  |
| 20 | Maswangi MRQ74 |  |
| 21 | Mat Chandu-468 |  |
| 22 | Merah Wangi |  |
| 23 | Mokwoo |  |
| 24 | MR12 |  |
| 25 | MR127 |  |
| 26 | MR142 |  |
| 27 | MR150 |  |
| 28 | MR185 |  |
| 29 | MR35 |  |
| 30 | MR56 |  |
| 31 | MR62 |  |
| 32 | Naylamp |  |
| 33 | Padi Rotan |  |
| 34 | Pulut Malaysia 1 |  |
| 35 | Pusa Basmathi |  |
| 36 | Q31 |  |
| 37 | Q70 |  |
| 38 | Q71 |  |
| 39 | Q72 |  |
| 40 | Q73 |  |
| 41 | Q74 |  |
| 42 | Q75 |  |
| 43 | QKK 16 |  |
| 44 | QKK 4-1 |  |
| 45 | RD3 |  |
| 46 | RP633E |  |
| 47 | Sadri |  |
| 48 | Siam Pilihan |  |
| 49 | Sona |  |
| 50 | Sri Malaysia 2 |  |
| 51 | Tainan 5 |  |
| 52 | Washabo |  |
| 53 | Y755 |  |
| 54 | Y756 |  |
| 55 | Basmati 370 |  |
| 56 | Kashmir Basmati |  |
| 57 | Apo |  |
| 58 | IR1561-228-3-3 |  |
| 59 | IR1561-243-5-6 |  |
| 60 | IR20 |  |
| 61 | IR2061-213-2-16 |  |
| 62 | IR2328-27-3-6 |  |
| 63 | IR2797-156-3 |  |
| 64 | IR28 |  |
| 65 | IR36 |  |
| 66 | IR38 |  |
| 67 | IR64 |  |
| 68 | IR77298-14-1-2-10 |  |
| 69 | IR828-28-1-1-1-2 |  |
| 70 | IR841-26-2 |  |
| 71 | IR841-5-1-2 |  |
| 72 | IRYN-VE82#202 |  |
| 73 | UPLRi7 |  |
| 74 | UPR-70-30-7 |  |
| 75 | UPT-4-25-1 |  |

**Table S9.** List of random and specific microsatellite markers group according to the chromosome number

| No. | Marker Name | Chromosome | Type |
| --- | --- | --- | --- |
| 1 | RM522 | 1 | Random marker |
| 2 | RM10488 | 1 | Random marker |
| 3 | RM582 | 1 | Random marker |
| 4 | RM113 | 1 | Random marker |
| 5 | RM129 | 1 | Random marker |
| 6 | RM513 | 1 | Random marker |
| 7 | RM315 | 1 | Random marker |
| 8 | RM104 | 1 | Random marker |
| 9 | RM12182 | 1 | Random marker |
| 10 | RM11943 | 1 | Specific *qDTY_1.1_* marker |
| 11 | RM431 | 1 | Specific *qDTY_1.1_* marker |
| 12 | RM12023 | 1 | Specific *qDTY_1.1_* marker |
| 13 | RM12091 | 1 | Specific *qDTY_1.1_* marker |
| 14 | RM12146 | 1 | Specific *qDTY_1.1_* marker |
| 15 | RM12233 | 1 | Specific *qDTY_1.1_* marker |
| 16 | RM212 | 1 | Specific *qDTY_1.2_* marker |
| 17 | RM3825 | 1 | Specific *qDTY_1.2_* marker |
| 18 | OSR17 | 2 | Random marker |
| 19 | RM12569 | 2 | Random marker |
| 20 | RM71 | 2 | Random marker |
| 21 | RM12727 | 2 | Random marker |
| 22 | RM12979 | 2 | Random marker |
| 23 | RM12992 | 2 | Random marker |
| 24 | RM324 | 2 | Random marker |
| 25 | RM262 | 2 | Random marker |
| 26 | RM523 | 2 | Random marker |
| 27 | RM327 | 2 | Specific *qDTY_2.1_* marker |
| 28 | RM521 | 2 | Specific *qDTY_2.1_* marker |
| 29 | RM3549 | 2 | Specific *qDTY_2.1_* marker |
| 30 | RM330 | 2 | Specific *qDTY_2.1_* marker |
| 31 | RM6374 | 2 | Specific *qDTY_2.1_* marker |
| 32 | RM13211 | 2 | Specific *qDTY_2.1_* marker |
| 33 | RM109 | 2 | Specific *qDTY_2.2_* marker |
| 34 | RM236 | 2 | Specific *qDTY_2.2_* marker |
| 35 | RM12460 | 2 | Specific *qDTY_2.2_* marker |
| 36 | RM279 | 2 | Specific *qDTY_2.2_* marker |
| 37 | RM555 | 2 | Specific *qDTY_2.3_* marker |
| 38 | RM263 | 2 | Specific *qDTY_2.3_* marker |
| 39 | RM573 | 2 | Specific *qDTY_2.3_* marker |
| 40 | RM15983 | 3 | Random marker |
| 41 | RM517 | 3 | Random marker |
| 42 | RM251 | 3 | Random marker |
| 43 | RM6817 | 3 | Random marker |
| 44 | RM16030 | 3 | Specific *qDTY_3.1_* marker |
| 45 | RM416 | 3 | Specific *qDTY_3.1_* marker |
| 46 | RM520 | 3 | Specific *qDTY_3.1_* marker |
| 47 | RM16672 | 4 | Random marker |
| 48 | RM142 | 4 | Random marker |
| 49 | RM17524 | 4 | Random marker |
| 50 | RM255 | 4 | Random marker |
| 51 | RM551 | 4 | Specific *qDTY_4.1_* marker |
| 52 | RM335 | 4 | Specific *qDTY_4.1_* marker |
| 53 | RM518 | 4 | Specific *qDTY_4.1_* marker |
| 54 | RM13 | 5 | Random marker |
| 55 | RM440 | 5 | Random marker |
| 56 | RM26 | 5 | Random marker |
| 57 | RM87 | 5 | Random marker |
| 58 | RM334 | 5 | Random marker |
| 59 | RM249 | 6 | Random marker |
| 60 | RM469 | 6 | Random marker |
| 61 | RM589 | 6 | Random marker |
| 62 | RM510 | 6 | Random marker |
| 63 | RM19637 | 6 | Random marker |
| 64 | RM541 | 6 | Random marker |
| 65 | RM20300 | 6 | Random marker |
| 66 | RM400 | 6 | Random marker |
| 67 | RM20776 | 7 | Random marker |
| 68 | RM180 | 7 | Random marker |
| 69 | RM542 | 7 | Random marker |
| 70 | RM320 | 7 | Random marker |
| 71 | RM21388 | 7 | Random marker |
| 72 | RM455 | 7 | Random marker |
| 73 | RM248 | 7 | Random marker |
| 74 | RM408 | 8 | Random marker |
| 75 | RM350 | 8 | Random marker |
| 76 | RM88 | 8 | Random marker |
| 77 | RM223 | 8 | Random marker |
| 78 | RM210 | 8 | Random marker |
| 79 | RM296 | 9 | Random marker |
| 80 | RM23680 | 9 | Random marker |
| 81 | RM444 | 9 | Random marker |
| 82 | RM219 | 9 | Random marker |
| 83 | RM524 | 9 | Random marker |
| 84 | RM108 | 9 | Random marker |
| 85 | RM553 | 9 | Random marker |
| 86 | RM566 | 9 | Specific *qDTY_9.1_* marker |
| 87 | RM24350 | 9 | Specific *qDTY_9.1_* marker |
| 88 | RM24390 | 9 | Specific *qDTY_9.1_* marker |
| 89 | RM24421 | 9 | Specific *qDTY_9.1_* marker |
| 90 | RM24932 | 10 | Random marker |
| 91 | RM222 | 10 | Random marker |
| 92 | RM311 | 10 | Random marker |
| 93 | RM25185 | 10 | Random marker |
| 94 | RM271 | 10 | Random marker |
| 95 | RM304 | 10 | Random marker |
| 96 | RM258 | 10 | Specific *qDTY_10.1_* marker |
| 97 | RM171 | 10 | Specific *qDTY_10.1_* marker |
| 98 | RM25694 | 10 | Specific *qDTY_10.1_* marker |
| 99 | RM590 | 10 | Specific *qDTY_10.1_* marker |
| 100 | RM286 | 11 | Random marker |
| 101 | RM552 | 11 | Random marker |
| 102 | RM441 | 11 | Random marker |
| 103 | RM287 | 11 | Random marker |
| 104 | RM229 | 11 | Random marker |
| 105 | RM21 | 11 | Random marker |
| 106 | RM224 | 11 | Random marker |
| 107 | RM519 | 12 | Random marker |
| 108 | RM28076 | 12 | Specific *qDTY_12.1_* marker |
| 109 | RM28083 | 12 | Specific *qDTY_12.1_* marker |
| 110 | RM28089 | 12 | Specific *qDTY_12.1_* marker |
| 111 | RM28099 | 12 | Specific *qDTY_12.1_* marker |
| 112 | Indel8 | 12 | Specific *qDTY_12.1_* marker |
| 113 | RM28130 | 12 | Specific *qDTY_12.1_* marker |
| 114 | RM1261 | 12 | Specific *qDTY_12.1_* marker |
| 115 | RM28166 | 12 | Specific *qDTY_12.1_* marker |
| 116 | CG29430 | 12 | Specific *qDTY_12.1_* marker |
| 117 | RM491 | 12 | Specific *qDTY_12.1_* marker |
| 118 | RM28048 | 12 | Specific *qDTY_12.1_* marker |
| 119 | RM511 | 12 | Specific *qDTY_12.1_* marker |
